# Physicians' perception of childhood asthma in Turkey: more appropriate practice among female physicians

**DOI:** 10.1186/1472-6963-8-155

**Published:** 2008-07-23

**Authors:** Ozge Uysal Soyer, Ersoy Civelek, Bulent E Sekerel

**Affiliations:** 1 Hacettepe University Faculty of Medicine, Pediatric Allergy and Asthma Unit 06100 Ankara, Turkey

## Abstract

**Background:**

Low levels of asthma control worldwide point to the possibility of sub-optimal management; therefore, documentation of physicians' perception is critical for future interventions. Our aim was to examine self-reported management abilities of Turkish physicians dealing with children with asthma, document the factors affecting appropriate decisions and compare the results with those of a previous survey.

**Methods:**

Physicians were surveyed via a questionnaire aimed to document self-perceived asthma knowledge and attitudes in asthma management.

**Results:**

The majority of physicians were male (63%) and examined 234 ± 9 patients per week. Infrequent use of objective parameters in asthma diagnosis and attack severity assessment was reported and most preferred nebulized corticosteroids to the systemic form in acute asthma. Even though self-perceived overall asthma knowledge did not differ between genders (p = 0.098), male physicians scored higher than females for inhaled steroids for acute asthma (2.8 ± 0.12 vs 2.17 ± 0.2, respectively, p = 0.007), while female physicians recorded more frequent use of inhaled steroids for chronic asthma (3.72 ± 0.08 vs 3.43 ± 0.07, respectively, p = 0.006). Female physicians' scoring for "symptom control" as the main aim of asthma management was higher than that of their male counterparts (3.88 ± 0.04 vs 3.65 ± 0.06, respectively, p = 0.002).

**Conclusion:**

Although there were some discrepancies between guidelines and clinical practice, most applications of Turkish physicians dealing with children with asthma were appropriate. Interestingly, when scores of female versus male physicians were compared, it can be suggested that female physicians have a more appropriate perception of asthma, indicating a significant contribution of gender-related factors in clinical attitudes and beliefs.

## Background

Asthma is a serious health problem, and the prevalence and societal burden of asthma are compelling, as well as disturbing, especially given the availability of effective evidence-based treatment [1]. Appropriate clinical practice of the physician is one of the most critical points in asthma management.

In our previous report in 2003, we documented that even though Turkish physicians' opinions generally appeared to be in agreement with the guidelines, there were certain gaps in clinical practice in terms of using objective parameters to assess severity, frequent use of nebulized corticosteroids for acute asthma and use of leukotriene modifiers as the first-line therapy [2]. These findings were further supported by the low control levels determined in a population-based study [3]. Various barriers exist for a successful integration of evidence-based medicine into clinical practice. Some relate to attitudes and beliefs held by patients, while others relate to the doctors' attitudes, personal experience and knowledge, national health care systems, economies and medical traditions [4,5]. Inappropriate diagnosis, pharmacotherapy and follow-up in asthma may lead to increased morbidity and mortality, as well as increased use of health care resources. Understanding the level of management ability of physicians may guide interventions to improve quality of care and patient outcomes. After the 2003 survey, a number of training activities were implemented by national allergy and respiratory societies focusing on the documented gaps, including meetings, courses and informational booklets and books, in an effort to increase physicians' knowledge about asthma. This questionnaire-based study, the second of such surveys, aimed to examine self-reported management abilities of Turkish physicians dealing with children with asthma in 2006, and documents the factors affecting appropriate decisions. It also addresses trends over time by comparing current results with those from the 2003 study.

## Methods

As in the previous study, this investigation focused on a group of physicians, mainly pediatricians, attending a pediatric asthma meeting sponsored by a pharmaceutical firm (Merck Sharp & Dohme, Turkey), held in November 2006. Physicians were invited by company because they were dealing with children with asthma (namely, those for whom asthma drugs constituted a majority of their prescriptions and for whom asthmatics constituted the majority of their patient spectrum). Questionnaires were distributed during the morning meeting and collected at the end of the scientific sessions.

The questionnaire was identical to the tool used in our previous study and was based on the information in guidelines [2]. The questionnaire was prepared by the authors and was not connected in any way with the industry. Briefly, the survey consists of questions about demographic factors, self-perception of asthma knowledge, parameters used for asthma diagnosis and estimation of acute attack severity, preference of medications for acute and chronic asthma treatment, and goals of long-term management [see Additional file 1]. All of the questions were prepared as statements and answered on a 5-point scale with minimum and maximum scores of 0 and 4 reflecting degree of physician agreement, with 4 denoting "total agreement". The results were evaluated only by the authors and were not shared with the pharmaceutical company.

### Analysis

The current and previous surveys and gender discrepancies were compared using independent samples t-test. Pearson correlation analysis was done to evaluate correlations. A value of P ≤ 0.05 was considered statistically significant.

## Results

### Study population

Of the 254 physicians attending the meeting, 165 (64.9%) responded to the questionnaire and all surveys were eligible for analysis. Because no item had missing responses for more than 5% of respondents, we omitted the number of missing responses for each item from data tables. Table 1 compares respondent characteristics for both the 2003 and present surveys. The majority of physicians were male (63%) and examined 234 ± 9 patients per week, including 42 ± 4 children with asthma.

**Table 1:** Characteristics of the study groups

Characteristics	2003 Survey	2006 Survey
	(n = 126)	(n = 165)
Sex (male/female)	85/41	104/61
Age	39.3 ± 0.6*	41.5 ± 0.6*
Duration of experience (yr)	15.2 ± 0.6*	16.6 ± 0.7*
Number of patients per week	253 ± 14*	234 ± 9*
Asthma patients per week	40.2 ± 4.5*	42.2 ± 3.5*
Speciality n (%)		
Pediatrician	105 (83.2)	157 (95.1)
Pediatrician + allergist	11 (8.8)	8 (4.9)
General practitioner	5 (4)	
Family physician	5 (4)	
Primary work place n (%)		
Hospital	84 (66.7)	98 (56.3)
Training hospital	11 (8.7)	18 (10.3)
University	14 (11.1)	15 (8.6)
Outpatient clinic	17 (13.5)	34 (19.5)

* Mean ± SEM

### Self-reported knowledge

Physicians' self-perception of asthma knowledge was questioned under four subcategories (overall, diagnosis, acute asthma and long-term management), and the highest score (3.16 ± 0.05) was determined for acute asthma management and the lowest (2.89 ± 0.06) for chronic asthma management. Though this trend was in agreement with the previous survey, previous scores were lower than those determined in the current survey (p ≤ 0.05, for each item).

### Self-reported diagnostic ability

Physicians reported "recurrent wheezing" (3.75 ± 0.039) and "reversibility with bronchodilator on lung function test" (3.65 ± 0.049) as the most important findings for asthma diagnosis, as in the previous survey. They disagreed with "Asthma diagnosis is difficult in absence of skin tests for atopy and lung function tests" (1.56 ± 0.08 and 1.74 ± 0.08, respectively). There was no statistical difference between the previous and current survey in terms of self-reported diagnostic ability parameters.

### Self-reported attack management

In the present survey, physicians reported that acute asthma attack severity was assessed by the presence of dyspnea (3.75 ± 0.04), retractions and wheezing (3.67 ± 0.043, for both). Objective parameters like lung function tests (3.1 ± 0.07), oxygen saturation (3.34 ± 0.06), and blood gases (3.01 ± 0.08) received relatively lower scores. Though short-acting β2 agonists (3.71 ± 0.05) were the treatment of choice for acute asthma, systemic and inhaled corticosteroids received similar scores (2.69 ± 0.09 and 2.57 ± 0.01, respectively) for the treatment of attack. Another interesting finding was that nebulized corticosteroids (2.78 ± 0.09) were preferred to systemic corticosteroids (2.69 ± 0.09), though the difference did not reach statistical significance (p > 0.05). These scores were comparable to those from the previous study; however, use of theophylline (0.97 ± 0.08) and ipratropium bromide (1.31 ± 0.1) received lower scores than in the previous study (p < 0.01).

### Self-reported chronic management

Asthma severity (3.65 ± 0.05) was scored as the most important parameter in treatment selection and inhaled corticosteroids received the highest grade (3.54 ± 0.06), whereas leukotriene receptor antagonists (LTRA) received a comparable score (3.4 ± 0.06). Interestingly, the scores for the use of LTRA were higher than in the previous survey (3.13 ± 0.08) (p < 0.01). Physicians' attitudes toward treatment goals revealed "control of symptoms" as the leading goal (3.74 ± 0.04), as in the previous survey (3.77 ± 0.04) (p = 0.5). However, the statement, "my treatment aims to achieve best lung function test", scored lower than in the previous survey (3.03 ± 0.07 and 3.31 ± 0.07, respectively) (p < 0.01).

### Factors affecting preferences

**Figure 1:**
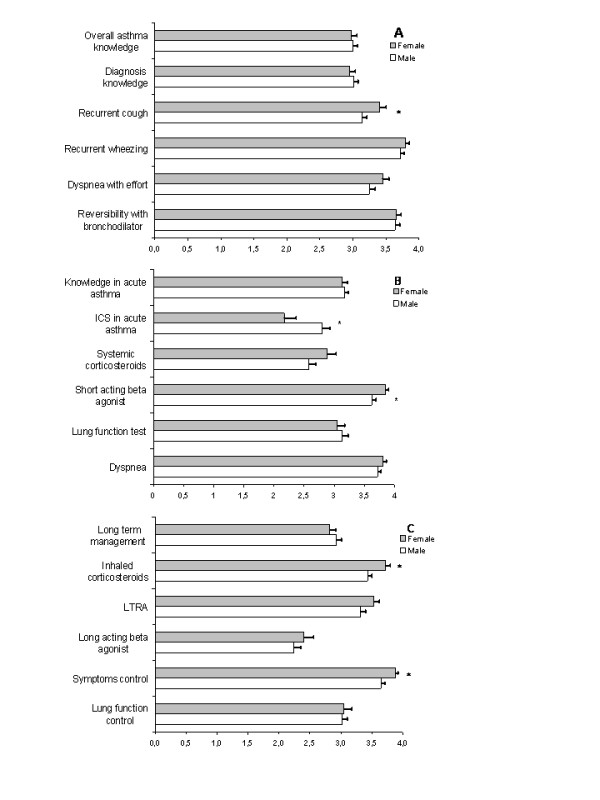
**Gender influence on attitudes of asthma management.** A. Self perception of asthma knowledge and diagnosis. B. Assessment and management of acute asthma. C. Management of chronic asthma. *: p < 0.05.

In order to examine the characteristics leading to more appropriate management, a number of comparisons were made for age, gender, number of seen patients/asthmatics per week, localization of practice (rural vs urban) and the duration of experience. Gender and age showed consistent statistical significance; no statistical significance was determined for the remaining parameters (data not shown). Male and female physicians were comparable with respect to age (42.47 ± 0.81 vs 39.9 ± 1 years, respectively, p = 0.051) and duration of experience (17.27 ± 0.81 vs 15.46 ± 1.09, respectively, p > 0.05), but females examined fewer patients per month (203 ± 14 vs 253 ± 12, respectively, p = 0.022). Even though self-perception of overall asthma knowledge did not differ between genders (p = 0.098), impact of gender on physician attitudes was remarkable. Female physicians more frequently noticed recurrent cough as an important symptom indicating the diagnosis of asthma than males (3.4 ± 0.1 vs 3.14 ± 0.07, respectively, p = 0.039). As expected, both groups evaluated short-acting beta 2 agonists as the most useful drug in acute asthma treatment; however, females scored short-acting beta 2 agonists higher (3.85 ± 0.06 vs 3.62 ± 0.07, respectively, p = 0.01). Although the groups had almost similar scores for the effectiveness of systemic corticosteroid treatment in acute asthma (males: 2.58 ± 0.11 vs females: 2.88 ± 0.15, p > 0.11), male physicians scored inhaled steroids higher than females (2.8 ± 0.12 vs 2.17 ± 0.2, respectively, p = 0.007). Though inhaled corticosteroids were the drug of choice for chronic asthma management in both groups, female physicians recorded more frequent use (3.72 ± 0.08 vs 3.43 ± 0.07, respectively, p = 0.006). There were no differences between male and female physicians' preferences for LTRA (3.32 ± 0.08 vs 3.53 ± 0.09, respectively, p = 0.077) and long-acting β agonists (2.24 ± 0.11 vs 2.4 ± 0.16, p = 0.4) usage for long-term treatment of asthma. The concept of symptom control was affirmed as the main aim of asthma management by both groups; however, female physicians scored higher than males (3.88 ± 0.04 vs 3.65 ± 0.06, respectively, p = 0.002) (Figure 1).

Correlation analysis revealed that with increasing age, physicians assigned a lesser degree of significance to "obstruction on lung function tests" (p = 0.004, r_s _= 0.229) and "reversibility with bronchodilator on lung function tests" (p = 0.006, r_s _= 0.219) for diagnosis of asthma, and preferred to use treatments with fewer side effects (p = 0.003, r_s _= 0.238). Clinician age was negatively and weakly correlated with short-acting beta agonist use for acute asthma attack (p = 0.003, r_s _= 0.241).

As analysis of the predictors of appropriate decision-making was not performed in the previous survey, a retrospective analysis was conducted to investigate the influence of gender on physicians' attitudes. Female physicians supported their diagnosis of asthma with the idea of "concomitant allergic diseases" more than males (3.63 ± 0.09 vs 3.46 ± 0.09, respectively, p = 0.015) and they agreed with "Asthma diagnosis is challenging during infancy" (3.48 ± 0.12 vs 2.9 ± 0.12, respectively, p = 0.001). Furthermore, females prescribed systemic corticosteroids more than males in treatment of acute asthma (3.24 ± 0.13 vs 2.81 ± 0.13, respectively, p = 0.019). The statement "Asthma severity is an important criterion for treatment selection" was preferred by females more than males (3.24 ± 0.13 vs 2.81 ± 0.13, respectively, p = 0.019).

## Discussion

In this questionnaire-based study, we showed that though there have been a number of minor improvements in the self-recorded practices of Turkish physicians when compared to the previous survey, there are still a number of inappropriate practices in terms of acute asthma management. More interestingly, we found that gender plays an important role in decision-making. The lack of any major change in physicians' practice suggests the ineffectiveness of the many continuing medical education programs conducted during the last four years and indicates the necessity of other instruments, such as case studies, real-life scenarios, campaigns, and audiovisual resources.

Available data suggest that asthma continues to be an important health problem worldwide. Given the attention paid to this condition, the contributing factors for diagnosis and management of asthma are important. When studying differences in health, behavior and attitudes, it is generally not possible to distinguish between what is biological versus social in origin. Gender views women and men from a psychosocial and cultural perspective that is socially constructed not biologically ordained [6]. In this study, male and female physicians were comparable in age, years of professional experience and level of self-perception of their overall asthma knowledge; nevertheless, differences were observed in some of their attitudes towards asthma diagnosis and management. Female physicians tend to display more patient-centered behavior, are more concerned about emotional and social aspects of health, and are more interested in a patient's input and partnership [7]. We found that female pediatricians examined fewer children per week so that they could spend more time with their patients in order to facilitate better listening and a more careful evaluation. In Turkey, part-time work based on gender differences is not available; male and female physicians should work equal hours.

Female physicians used short-acting beta 2 agonists more frequently than males for acute asthma attacks. The most striking finding was that male physicians used inhaled steroids more than females for acute asthma, which is not in accordance with asthma guidelines. Four years ago, females used systemic corticosteroids more than males for acute asthma. Women are considered careful and hard-working, and may follow the innovations in medicine mindfully and more easily change their management routines according to new guidelines [8]. This fosters greater patient trust in female physicians. Shah and Ogden showed that with respect to expected patient behavior, respondents rated female doctors higher than males in terms of having faith in their diagnosis, being more likely to accept their advice and being more likely to choose to see that doctor [9]. Again, inhaled steroids, the cornerstone of chronic asthma therapy, were rated higher by female physicians despite physicians' expressions of specific concerns about corticosteroid use when queried. In management guidelines, the control of asthma is the goal of disease management, which was confirmed by female physicians more than males. In the previous survey, females also scored higher regarding asthma severity as an important guide to antiinflammatory drug selection. The fact that this study was not intended to focus on gender differences, thus minimizing the risk of reinforcing gender-related dichotomies or of producing individual gender bias, can be considered one of its strengths.

In our study, we found that older and more experienced physicians were less likely to use objective parameters like lung function tests for asthma diagnosis and beta 2 agonists for asthma attacks. This may be attributed to the deep-rooted attitudes of older clinicians, who are resistant to change their practice in accordance with present-day guidelines and who are highly confident in their knowledge based on their long years of medical experience. Older pediatricians give clinically oriented decisions while younger pediatricians are more dependent on laboratory examinations.

Changing diagnosis routines is important in changing clinical practice. Compared to our previous study, there is still a lack of use of objective parameters in both asthma diagnosis and severity assessment of asthma attack. In the research by Finkelstein *et al.*, it was stated that 12% and 3% of pediatricians in the US used spirometry and skin or radioallergosorbent testing always or most of the time for initial work-up, respectively [10]. However, in our study, "reversibility with bronchodilator on lung function test" scored higher for asthma diagnosis, while skin test positivity had one of the lowest scores. Our physician group failed to utilize objective disease severity monitoring as often as suggested by the guidelines. Although Turkey is a developing country, spirometers and pulse oximeters are inexpensive tools and if physicians wish to use them in their practice, the Ministry of Health will provide them. Therefore, lack of availability cannot be considered a reason for their not being used in clinical practice.

Providing doctors with knowledge on how to treat disease in accordance with research evidence and guideline recommendations seldom changes the way doctors prescribe drugs [11]. Despite the many educational programs in large cities regarding asthma, 5–6 national congresses/year including asthma sessions, and distribution of national asthma guidelines to all pediatricians during the last four years, which stressed the importance of childhood asthma management, no decline was observed in the use of inhaled steroids in acute asthma attack. Furthermore, the new guidelines were discussed in a significant number of meetings. Unfortunately, in Turkey, admissions to meetings are voluntary, and there is no credit system to facilitate participation in these meetings. For long-term asthma management, inhaled corticosteroids and LTRA were the most frequently preferred drugs. In Turkey, the social security system covers the cost of all anti-inflammatory drugs and bronchodilators in the treatment of asthma, and all are readily available throughout the country. When compared to our previous survey, use of LTRA had increased significantly. The advent of this new class of agents offers the clinician a new opportunity to treat his or her patients with drugs other than steroids, given the concerns about their safety and adverse effects [10], and children might be expected to be more compliant in taking once-daily oral medications [12].

Participants of this study were a particularly motivated group, willing to learn from seminars and educational programs. These doctors might be somewhat more interested and involved in keeping up to date in comparison with their colleagues. Since this study was based on responses to a survey, it might not reflect what physicians actually do in their daily practice. We are aware that, on a scale of 5, some of the differences determined were small and that statistical significance does not always indicate clinical significance. Nevertheless, since even these minor changes may have important clinical implications in terms of early and correct diagnosis, improved control levels, and appropriate assessment tools, we feel the difference should at least be considered.

## Conclusion

In conclusion, we showed that although there were some discrepancies between guidelines and clinical practice in terms of objective parameters and acute asthma management, most applications were appropriate. Unfortunately, we were unable to show a major improvement in asthma practices in spite of the number of activities held during the interim, indicating the need for alternative instruments to improve management. More interestingly, in the present and previous surveys, female physicians demonstrated better asthma management than males, the reasons for which are not defined and can only be speculated. Determining gender-related factors underlying decision-making should be a goal of future studies.

## Competing interests

• The meeting was sponsored by Merck Sharp & Dohme.

• The authors declare that they have no financial or non-financial competing interests with Merck Sharp & Dohme or any other pharmaceutical company.

## Authors' contributions

*OUS *participated in the development of the protocol and analytic framework of the study, had primary responsibility for data analysis, and prepared the manuscript with *BES*. *EC *participated in the development of the protocol and analytic framework of the study, and contributed to preparation of the manuscript. *BES *had primary responsibility for protocol development and outcome assessment, contributed to data analysis, and prepared the manuscript with *OU*S. *OUS, EC *and *BES *read and approved the final manuscript.

## Pre-publication history

The pre-publication history for this paper can be accessed here:



## Supplementary Material

Additional file 1 Questionnaire, key questions. The data provided represent the key questions of the questionnaire applied to the physicians included in the study.
Click here for file
