# Supplementary material for: Physicians' perception of childhood asthma in Turkey: more appropriate practice among female physicians
Source: BMC Health Serv Res. 2008 Jul 23;8:155. doi: 10.1186/1472-6963-8-155 (PMC2488342; doi:10.1186/1472-6963-8-155)
Supplement: Additional file 1 — Questionnaire, key questions. The data provided represent the key questions of the questionnaire applied to the physicians included in the study. [file 1472-6963-8-155-S1.doc]

**Questionnaire, key questions**

1. Age:………………………………………………………………..
2. Gender:………...………………………………………………….
3. How many years have you been working as a doctor?.........…….
4. Your specialty:……………………………………………………..
5. Your place of work?..........................................................................
6. Percentage of children with asthma in your daily practice %..................
7. Your asthma-related knowledge (4=very good, 0=insufficient):

Overall asthma

Asthma diagnosis

Acute asthma attack

Long-term asthma management

1. Parameters for asthma diagnosis (4=very important, 0=unimportant):

Recurrent dry cough

Recurrent wheezing

Dyspnea

Dyspnea on exertion

Increased symptoms during nighttime

Concomitant allergic disease other than asthma

Family history of atopic disease

Obstruction in lung function tests

Reversibility on lung function test

Elevated eosinophil count

Elevated serum IgE level

Allergy detected by skin prick tests/specific IgE

1. Statements for diagnosis of asthma (4=I agree completely, 0=I disagree completely):

Asthma diagnosis is challenging during infancy.

Asthma diagnosis is difficult in absence of skin tests for atopy and lung function tests.

The diagnosis of asthma is difficult without excluding other diseases.

1. Parameters for assessment of asthma attack severity (4=very important, 0=unimportant):

General condition

Vital signs

Skin color

Retractions

Dyspnea

Wheezing

Auscultation

Oxygen saturation

Blood gases

Lung function tests

Chest X-ray

1. Drug preference for the treatment of asthma attacks (4=often, 0=never):

Short-acting beta agonists

Long-acting beta agonists

Inhaled corticosteroids

Nebulized corticosteroids

Systemic corticosteroids

Ipratropium bromide

Theophylline

Leukotriene receptor antagonists

Adrenalin

1. Drug preference for chronic asthma management (4=often, 0=never):

Short-acting beta agonists

Long-acting beta agonists

Inhaled corticosteroids

Nebulized corticosteroids

Systemic corticosteroids

Sodium cromoglycate

Nedocromil

Leukotriene receptor antagonists

Ketotifen

1. Statements for asthma management (4=I agree completely, 0= I disagree completely):

My treatment aims to achieve patient and family satisfaction with asthma control level.

I generally aim for optimum control with minimum drugs.

I generally use intensive treatment to achieve best control.

My treatment aims to achieve best lung function test.
